# MRI-based tumor shrinkage patterns after early neoadjuvant therapy in breast cancer: correlation with molecular subtypes and pathological response after therapy

**DOI:** 10.1186/s13058-024-01781-1

**Published:** 2024-02-12

**Authors:** Mengfan Wang, Siyao Du, Si Gao, Ruimeng Zhao, Shasha Liu, Wenhong Jiang, Can Peng, Ruimei Chai, Lina Zhang

**Affiliations:** Department of Radiology, The First Hospital of China Medical University, Nanjing North Street 155, Shenyang, 110001 Liaoning Province China

**Keywords:** Breast cancer, Neoadjuvant therapy, Magnetic resonance imaging, Tumor shrinkage patterns, Pathologic complete response

## Abstract

**Background:**

MRI-based tumor shrinkage patterns (TSP) after neoadjuvant therapy (NAT) have been associated with pathological response. However, the understanding of TSP after early NAT remains limited. We aimed to analyze the relationship between TSP after early NAT and pathological response after therapy in different molecular subtypes.

**Methods:**

We prospectively enrolled participants with invasive ductal breast cancers who received NAT and performed pretreatment DCE-MRI from September 2020 to August 2022. Early-stage MRIs were performed after the first (1st-MRI) and/or second (2nd-MRI) cycle of NAT. Tumor shrinkage patterns were categorized into four groups: concentric shrinkage, diffuse decrease (DD), decrease of intensity only (DIO), and stable disease (SD). Logistic regression analysis was performed to identify independent variables associated with pathologic complete response (pCR), and stratified analysis according to tumor hormone receptor (HR)/human epidermal growth factor receptor 2 (HER2) disease subtype.

**Results:**

344 participants (mean age: 50 years, 113/345 [33%] pCR) with 345 tumors (1 bilateral) had evaluable 1st-MRI or 2nd-MRI to comprise the primary analysis cohort, of which 244 participants with 245 tumors had evaluable 1st-MRI (82/245 [33%] pCR) and 206 participants with 207 tumors had evaluable 2nd-MRI (69/207 [33%] pCR) to comprise the 1st- and 2nd-timepoint subgroup analysis cohorts, respectively. In the primary analysis, multivariate analysis showed that early DD pattern (OR = 12.08; 95% CI 3.34–43.75; *p* < 0.001) predicted pCR independently of the change in tumor size (OR = 1.37; 95% CI 0.94–2.01; *p* = 0.106) in HR^+^/HER2^−^ subtype, and the change in tumor size was a strong pCR predictor in HER2^+^ (OR = 1.61; 95% CI 1.22–2.13; *p* = 0.001) and triple-negative breast cancer (TNBC, OR = 1.61; 95% CI 1.22–2.11; *p* = 0.001). Compared with the change in tumor size, the SD pattern achieved a higher negative predictive value in HER2^+^ and TNBC. The statistical significance of complete 1st-timepoint subgroup analysis was consistent with the primary analysis.

**Conclusion:**

The diffuse decrease pattern in HR^+^/HER2^−^ subtype and stable disease in HER2^+^ and TNBC after early NAT could serve as additional straightforward and comprehensible indicators of treatment response.

*Trial registration*: Trial registration at https://www.chictr.org.cn/. Registration number: ChiCTR2000038578, registered September 24, 2020.

**Supplementary Information:**

The online version contains supplementary material available at 10.1186/s13058-024-01781-1.

## Introduction

Neoadjuvant therapy (NAT) has become the important treatment for locally advanced breast cancers, and patients who achieve pathologic complete response (pCR) after NAT demonstrate improved prognosis and survival [1–3]. However, due to the high heterogeneity of breast cancer, the efficacy of NAT varies significantly among individuals [4]. Early monitoring NAT response of tumors is important for timely adjustment of treatment regimens to optimize efficacy, avoid unnecessary adverse effects and increase disease-free survival [5, 6].

Dynamic contrast enhanced (DCE) MRI is a highly precise imaging technique that permits evaluation of a viable tumor before and after NAT by detecting changes in tumor vascularity [7, 8]. Response Evaluation Criteria in Solid Tumors (RECIST) 1.1 criteria [9] defines tumor response based on the decrease in the longest tumor diameter relative to the pretreatment baseline measurement. However, tumors exhibit various patterns of shrinkage as a result of intricate processes such as necrosis, fibrosis, inflammation, and other internal changes following NAT [10–12]. In breast cancer, the presence of diffuse nonmass enhancement on the pretreatment MRI or scattered foci within a fibrotic region on the posttreatment MRI poses a challenge to accurately predicting pCR using size measurements [13, 14].

Several studies have been conducted to investigate the relationship between tumor shrinkage patterns (TSP) and treatment response [11, 15]. It has been observed that concentric and fragmented shrinkage patterns are more commonly observed in patients achieving pCR, while stable disease is noted in those who do not achieve pCR during the middle stage and after NAT [16, 17]. Furthermore, analyses have demonstrated variations in TSP among different subtypes [18–20]. However, the understanding of TSP following early treatment (i.e., the first or second cycle of NAT) and their association with treatment response remains limited. Given that the alteration in tumor size following early treatment does not consistently provide reliable pCR prediction [21–23], we propose the hypothesis that early TSP may serve as an alternative imaging indicator for pCR prediction. This approach offers the advantage of being easily interpretable and applicable in clinical settings.

In this prospective study, we performed longitudinal breast DCE-MRI before and after early NAT to describe TSP and investigate its role as a predictor of therapeutic response. Since the NAT regimens and pCR rate differed among different molecular subtypes, we performed stratified analysis according to molecular subtype.

## Materials and methods

### Participants

In this prospective, single-center, observational study, 362 participants with primary invasive ductal carcinoma who performed pretreatment DCE-MRI were enrolled. Participants eligible for our study included women with invasive breast tumors 1.0 cm or larger at imaging examination who were planning to undergo NAT. Participants with evidence of distant metastasis or progressive diseases during NAT that resulted in changing the initial NAT regimen or surgery cancellation were excluded. Our institutional review board approved this study and each participant provided written informed consent.

This study involved conducting DCE-MRI examinations at three specific timepoints during NAT, including pretreatment (referred to as Pre-MRI), after the first cycle of NAT (referred to as 1st-MRI), and/or after the second cycle of NAT (referred to as 2nd-MRI). The decision to perform Pre-MRI and 2nd-MRI was made by clinicians [24], while 1st-MRI was additionally recommended by clinicians for earlier efficacy evaluation, and its execution was contingent upon the individual preferences of participants.

**Fig. 1:**
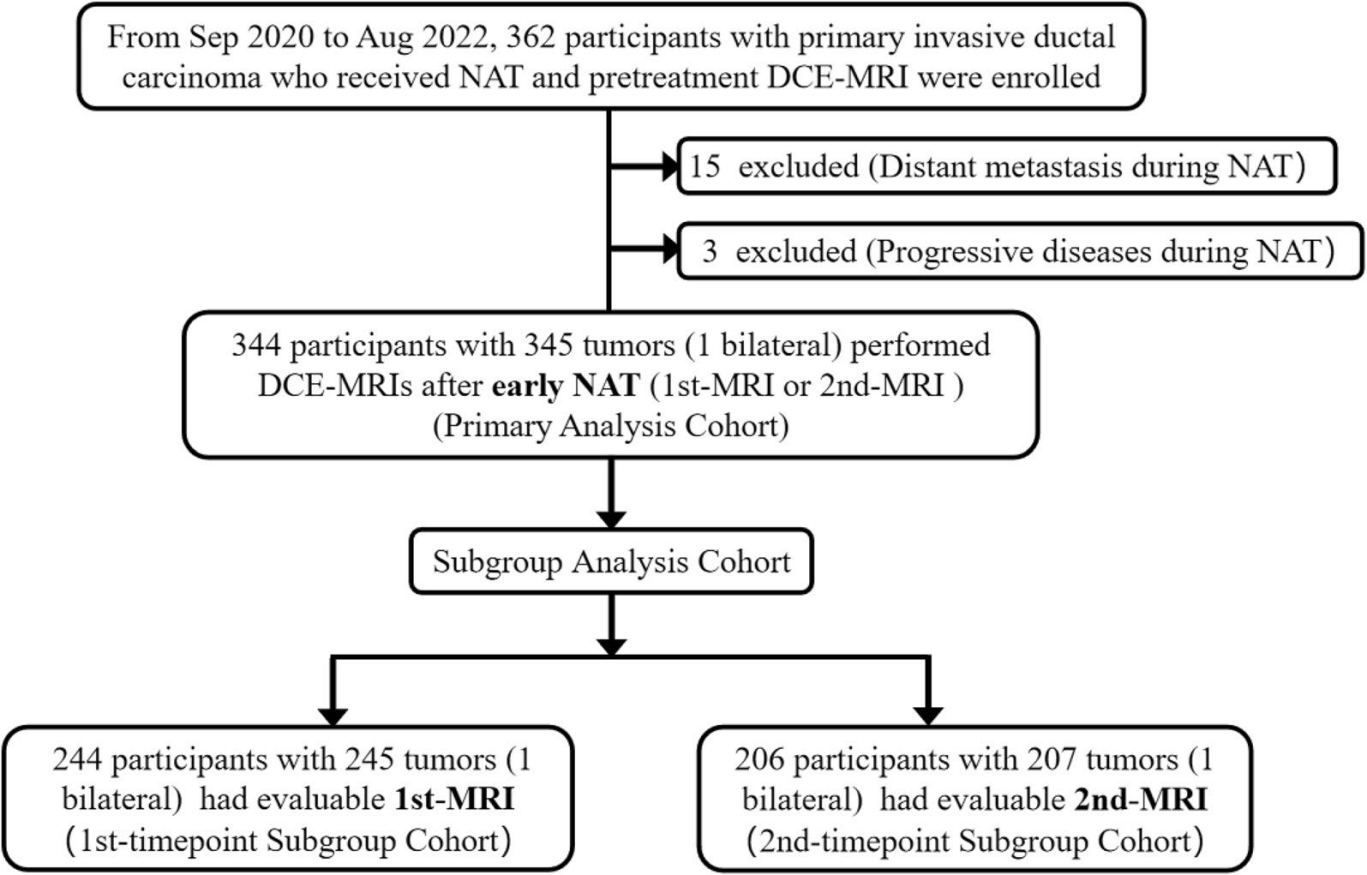
Flowchart of study participants

Participants who performed DCE-MRI before and after early NAT (either 1st-MRI or 2nd-MRI usable) were used as the primary analysis cohort to describe TSP and investigate the value as an early pCR predictor. The primary analysis was an “intention-to-diagnose analysis” based on the total cohort of randomized participants. If a participant performed both 1st-MRI and 2nd-MRI, 2nd-MRI data of the participant were used for primary analysis. To further analyze TSP after 1st-MRI or 2nd-MRI, we conducted a subgroup analysis to determine the earliest timepoint at which TSP worked. The subgroup analysis was an “per-protocol analysis” based on complete 1st-MRI or 2nd-MRI data (referred to as 1st-timepoint and 2nd-timepoint subgroup analysis, respectively). Participants enrollment flowchart and the cohorts for the primary analysis and subgroup analysis are shown in Fig. 1.

### Treatment protocol

All participants received standard six or eight cycles of NAT before surgery according to the National Comprehensive Cancer Network guideline [7]. The NAT regimens were based on anthracycline, taxane, or both anthracycline and taxane. For human epidermal growth factor receptor 2 (HER2)-positive tumors, anti-HER2 targeted trastuzumab (H) or trastuzumab + pertuzumab (HP) were added to the chemotherapy drugs.

### Imaging analysis

All breast MRI examinations were performed on a 3.0T MR scanner (SIGNA™ Pioneer, GE Healthcare, Milwaukee, WI, USA) in the prone position using a dedicated 8-channel phased-array breast coil. T1-weighted (T1W) DCE-MRI sequence in the axial plane with temporal resolution of 19.4 s was obtained using three-dimensional (3D) DISCO and fat suppression technique. The scanning parameters were as follows: repetition time/echo time (TR/TE) = 4.9/1.7 ms, flip angle = 10°, field of view (FOV) = 360 × 360 mm, acquisition matrix = 256 × 256, slice thickness/gap = 1.4 mm, number of sections = 116/phase, acceleration factors = 2. After the pre-contrast scanning followed by a pause of 20 s, the contrast agent was injected intravenously as a bolus (0.1 mmol/kg body weight) by a power injector at 2 mL/s followed by a 20 mL saline flush. Subsequently, 16–20 phase post-contrast images were acquired. Additional imaging protocol details can be found in our previous publication [25].

**Fig. 2:**
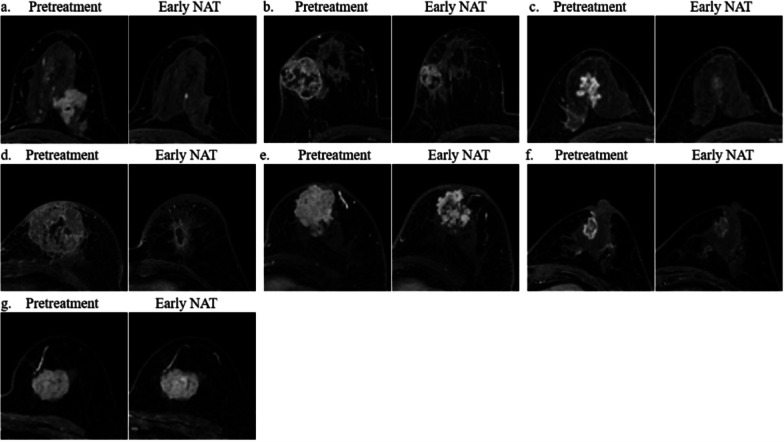
Shrinkage patterns of mass lesions. **a** Concentric shrinkage (CS): CS to small foci (pretreatment: a well demarcated 47 mm mass, early neoadjuvant therapy [NAT]: tumor size was significantly reduced with only residual enhancement foci < 5 mm), **b** CS: simple CS (pretreatment: a well demarcated 45 mm mass, early NAT: tumor size decreased to 32 mm without any morphological changes), **c** CS: CS plus decreased enhancement (pretreatment: an irregular 32 mm mass, early NAT: tumor size decreased to 25 mm with significantly reduced enhancement), **d** diffuse decrease (DD): CS with surrounding lesions (pretreatment: a 83 mm mass, early NAT: The tumor was distinctly CS with peripherally focal lesions), **e** DD: shrinkage with residual multinodular lesions (pretreatment: a 60 mm mass, early NAT: tumor splits into uniform fragments mixed with fibrous stroma) **f** decrease of intensity only (DIO) (pretreatment: an irregular 21 mm mass, early NAT: the degree of enhancement were obviously reduced but unchanged size) and **g** stable disease (SD) (pretreatment: a 35 mm mass, early NAT: no change)

**Fig. 3:**
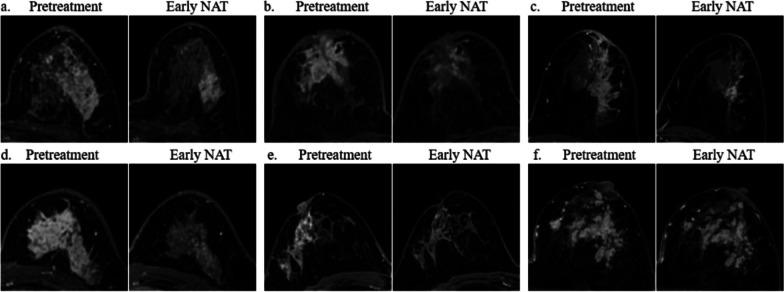
Shrinkage patterns of non-mass lesions. **a** concentric shrinkage (CS): simple CS (pretreatment: a regional 62 mm non-mass, early neoadjuvant therapy [NAT]: tumor size decreased to 27 mm without any morphological changes), **b** CS: CS plus decreased enhancement (pretreatment: a multiple regions 54 mm non-mass, early NAT: tumor size decreased to 44 mm with significantly reduced enhancement) **c** diffuse decrease (DD): CS with surrounding lesions (pretreatment: a segmental 100 mm non-mass, early NAT: the main lesion showed CS with peripheral focal lesions), **d** DD: shrinkage with residual multinodular lesions (pretreatment: a diffuse 75 mm non-mass, early NAT: tumor splits into uniform small fragments mixed with fibrous stroma), **e** decrease of intensity only (DIO) (pretreatment: a regional 60 mm non-mass, early NAT: the degree of enhancement was obviously reduced but unchanged size), **f** stable disease (SD) (pretreatment: a diffuse non-mass, early NAT: no changes). No CS to small foci non-mass lesions in our study

The assessment of TSP was conducted through a comprehensive analysis of the initial, peak and late post-contrast phases (specifically, the 5th, 7th and 16th post-contrast phases) of DISCO DCE-MRI according to the time intensity curve [11, 17]. We divided TSP into four groups based on Fukada et al.’s study [11]: concentric shrinkage (CS), diffuse decrease (DD), decrease of intensity only (DIO), and stable disease (SD). The CS pattern was further divided into three types: the simple CS, CS to small foci and CS plus decreased enhancement. The DD pattern was further divided into two types: concentric shrinkage with surrounding lesions, residual multinodular lesions (Figs. 2, 3). All image analyses were independently evaluated by two breast radiologists (W.M.F. and D.S.Y.), with 5 and 10 years of experience, respectively. In cases of inconsistent decisions, resolution was reached through consultation between two radiologists. If the two radiologists were unable to reach a decision after consultation, a third radiologist (Z.L.N., with 20 years of experience) made the final decision. They were blinded to tumor clinicopathological information.

For Pre-MRI, tumor maximum diameter was measured on the axial plane at peak phase. If multiple lesions were present, the largest tumor was selected as the targeted lesion. For follow-up images (1st-MRI or 2nd-MRI), the distance between the two farthest lesions was measured as the maximum diameter of the residual tumors for the DD pattern, while for the other patterns, the maximum diameter was measured consistently with the baseline. For the primary analysis, the tumor size before and after early NAT was recorded as D_pre_ and D_early_, and tumor size on 2nd-MRI was used as D_early_ for participants who performed both 1st-MRI and 2nd-MRI. The percentage changes (Δ%) in tumor size after early NAT was calculated using the following equation: ΔD_early_% = (D_pre _− D_early_)/D_pre_ × 100%. For subgroup analysis, tumor size measured on 1st-MRI and 2nd-MRI was recorded as D_1st_ and D_2nd_, respectively. The Δ% on 1st-MRI and 2nd-MRI was calculated using the following equation: ΔD_1st_% = (D_pre _− D_1st_)/D_pre_ × 100%, ΔD_2nd_ % = (D_pre _− D_2nd_)/D_pre_ × 100%. The mean value of tumor size measured by both readers was used for the final analysis. Additionally, tumor morphological and kinetic features were analyzed according to the 5th Ed. Breast Imaging Reporting and Data System (BI-RADS) lexicon [26].

### Histopathology

All patients received a core-needle biopsy guided by ultrasonography before NAT. The pathological specimens were viewed and diagnosed by a breast pathologist with more than 20 years of experience in breast pathologic examination. Immunohistochemistry (IHC) was performed for each patient to determine the baseline estrogen receptor (ER), progesterone receptor (PR), HER2 status, and Ki-67 index. According to ASCO guideline [27], the cutoff value for ER and PR was set at 1%, and the cutoff value for Ki67 was 20%. Regarding HER2 status, tumors with an IHC staining of 0 to 1+ were defined as HER2 negative and 3+ as HER2 positive. Fluorescence in situ hybridization was conducted when HER2 expression was detected as 2+ on IHC. A non-amplified FISH result denotes the HER2 status as negative, and an amplified result denotes the HER2 status as positive. Based on ER, PR, and HER2 status, the biological subtypes included the following: hormone receptor (HR)^+^/HER2^−^ (ER^+^ and/or PR^+^ and HER2^−^), HER2^+^ (HER2^+^ regardless of HR status) and triple-negative breast cancer (TNBC: ER^−^, PR^−^, and HER2^−^).

### Definition of histologic therapeutic effects

Postoperative pathological response was graded based on the Miller-Payne grading system [28]. pCR was defined as ypT0 or ypTis with no residual invasive tumor (Miller–Payne grade 5, residual ductal carcinoma in situ could be present). Patients with Miller-Payne grades 1 or 2 were classified into the nonresponse group (pNR), and patients with grades 3, 4, or 5 were in the response group (non-pNR) (Table 1). The histopathologic status of the axillary lymph nodes was not considered in pCR definition.

**Table 1:** Miller–Payne grading system

Miller–Payne grading	Explanation
Grade 1	No change or some alteration to individual malignant cells but no reduction in overall cellularity
Grade 2	A minor loss of tumor cells but overall cellularity still high; up to 30% loss
Grade 3	Between an estimated 30% and 90% reduction in tumor cells
Grade 4	A marked disappearance of tumor cells such that only small clusters or widely dispersed individual cells remain; more than 90% loss of tumor cells
Grade 5	No malignant cells identifiable in sections from the site of the tumor; only vascular fibroelastotic stroma remains often containing macrophages. However, ductal carcinoma in situ (DCIS) may be present

### Statistical analysis

Mann–Whitney and Chi-square (or Fisher’s exact) tests were used to compare the differences in clinicopathological and imaging features between the pCR and non-pCR groups (or pNR and non-pNR groups in HR^+^/HER2^−^ subtype). To compare TSP in different treatment response groups, the Chi-square test and Bonferroni correction for multiple comparisons were used, with a *p* value < 0.00833 (*p* < 0.05/6) considered statistically significant. The inter-reader agreement between both readers for TSP was calculated using Cohen’s Kappa (κ).

Clinicopathologic and imaging features potentially predictive for pCR were analyzed using binary logistic regression. Factors with a *p* value of < 0.10 on univariate logistic regression were entered into multivariate logistic regression and a *p* value < 0.05 was statistically significant. Performance for predicting pCR was assessed with the area under the receiver operating characteristic curve (AUC), accuracy, sensitivity, specificity, positive and negative predictive values (PPV and NPV). All analyses were performed using Statistical Package for the Social Sciences (SPSS, version 25.0, IBM Corporation, Armonk, NY, USA) and MedCalc (version 15.6.1).

## Results

### Participants characteristics

A total of 362 consecutive participants from September 2020 to August 2022 were enrolled in our study. Eighteen (5.0%) of 362 participants were excluded due to evidence of distant metastasis or progressive diseases during NAT. The remaining 344 participants with 345 tumors (1 bilateral, mean age: 50 years) who underwent DCE-MRI examinations after early NAT comprised the primary analysis cohort, which included 138 1st-MRI and 207 2nd-MRI examinations. For subgroup analysis, 244 of 344 participants (245 tumors) who had evaluable 1st-MRI and 206 of 344 participants (207 tumors) who had evaluable 2nd-MRI comprised the 1st- and 2nd-timepoint subgroup analysis cohorts, respectively (Fig. 1).

Baseline characteristics for the primary analysis cohort and two subgroup analysis cohorts are listed in Table 2. The most common molecular subtype was HR^+^/HER2^−^ (151/345, 44%) followed by HER2^+^ (123/345, 36%) and TNBC (71/ 345, 21%). After NAT, 113/345 (33%) achieved pCR. No significant difference was found in the primary analysis cohort versus the two subgroup analysis cohorts across all characteristics (Table 2, all *p* > 0.05).

**Table 2:** Participants characteristics

Characteristics	Primary analysis cohort	Subgroup analysis cohort			
		1st-timepoint subgroup		2nd-timepoint subgroup	
	n = 345^a^	n = 245	*p*	n = 207	*p*
Age (year), IQR	51 (42, 58)	51 (42, 58)	0.865	50 (41, 58)	0.427
Tumor size (mm), IQR	37 (27, 49)	36 (27, 49)	0.786	37 (28, 49)	0.965
Menopausal status			0.595		0.947
Premenopausal	174 (50)	129 (53)		105 (51)	
Postmenopausal	171 (50)	116 (47)		102 (49)	
TNM			0.726		0.839
IIA	64 (19)	49 (20)		33 (16)	
IIB	127 (37)	79 (32)		84 (41)	
IIIA	50 (15)	33 (14)		31 (15)	
IIIB	27 (7.8)	24 (10)		13 (6.3)	
IIIC	77 (22)	60 (25)		46 (22)	
Histologic grade			0.619		0.427
2	239 (69)	165 (67)		150 (73)	
3	106 (31)	80 (33)		57 (28)	
Molecular subtype			0.747		0.871
HR^+^/HER2^−^	151 (44)	100 (41)		94 (45)	
HER2^+^	123 (36)	94 (38)		74 (36)	
TNBC	71 (21)	51 (21)		39 (19)	
NAT regimen			0.763		0.943
Anthracycline-based	29 (8.4)	17 (7.1)		16 (7.7)	
Taxane-based	123 (36)	92 (38)		76 (37)	
Anthracycline and taxane-based	193 (56)	136 (56)		115 (56)	
Treatment response			0.855		0.888
pCR	113 (33)	82 (33)		69 (33)	
Non-pCR	232 (67)	163 (67)		138 (67)	
FGT			0.747		0.787
Scattered	70 (20)	51 (21)		39 (19)	
Heterogeneously	211 (61)	143 (58)		125 (60)	
Extremely dense	64 (19)	51 (21)		43 (21)	
BPE			0.860		0.404
Minimal	35 (10)	29 (12)		14 (6.8)	
Mild	192 (56)	139 (57)		113 (55)	
Moderate	88 (26)	58 (24)		56 (27)	
Marked	30 (8.7)	19 (7.8)		24 (12)	
Enhancement type			0.756		0.716
Mass	281 (81)	202 (82)		166 (80)	
Non-mass	64 (19)	43 (18)		41 (20)	
Multiplicity			0.521		0.190
Single lesion	129 (37)	98 (40)		66 (32)	
Multi-lesion	216 (63)	147 (60)		141 (68)	
Shape			0.491		0.595
Round or oval	68 (20)	54 (22)		37 (18)	
Irregular	277 (80)	191 (78)		170 (82)	
Margin			0.688		0.735
Circumscribed	24 (7.0)	15 (6.1)		16 (7.7)	
Not circumscribed	321 (93)	230 (94)		191 (92)	
Kinetics			0.962		0.896
Persistent	8 (2.3)	5 (2.0)		4 (1.9)	
Plateau	123 (36)	86 (35)		71 (34)	
Washout	214 (62)	154(63)		132 (64)	

Unless otherwise specified, data are numbers of participants, with percentages in parentheses

*p* values show the results of comparisons between the participants in the subgroup cohorts versus the participants in the primary analysis cohort

*pCR* pathologic complete response, *HER2* human epidermal growth factor receptor 2, *TNBC* triple-negative breast cancer, *HR* hormone receptor, *NAT* neoadjuvant therapy, *BPE* background parenchymal enhancement, *FGT* fibroglandular tissue

^a^345 MRI including 138 1st-MRI and 207 2nd-MRI

In the primary analysis cohort, pCR tended to present with high histologic grade, low D_early_ and large change in tumor size (*p* < 0.001). Molecular subtype and NAT regimen showed a significant association with pCR (*p* < 0.001). No significant difference was detected between participants with pCR and non-pCR in terms of age, baseline tumor size, menopausal status, clinical TNM stage and other MRI characteristics (Additional file 1: Table S1, all *p* > 0.05). The participants characteristics in subgroup analysis cohorts were consistent with those of the primary analysis cohort (Additional file 1: Table S2).

### Inter-reader agreement

The inter-reader agreement was considered almost perfect in the primary analysis (κ = 0.929), 1st-timepoint (κ = 0.942), and 2nd-timepoint (κ = 0.941) subgroup analysis cohorts. The detailed results for two readers are shown in Additional file 1: Tables S3 and S4.

### The primary analysis after early NAT

Table 3 shows the TSP for each molecular subtype in the primary analysis cohort. After early NAT, the CS pattern had the highest frequency in each molecular subtype (78/151 [52%] for HR^+^/HER2^−^, 67/123 [54%] for HER2^+^ and 45/71 [63%] for TNBC), with mainly simple CS pattern. The DD pattern had 29/151 (19%) in HR^+^/HER2^−^, 41/123 (33%) in HER2^+^ and 13/71 (18%) in TNBC. The SD pattern had 41/151 (27%) in HR^+^/HER2^−^, 12/123 (10%) in HER2^+^ and 13/71 (18%) in TNBC. The DIO pattern rarely appeared after early NAT, with only 3/151 (2.0%) in HR^+^/HER2^−^, 3/123 (2.4%) in HER2^+^ and 0/71 in TNBC.

**Table 3:** MRI-based tumor shrinkage patterns association with pCR according to different molecular subtypes in the primary analysis cohort

Shrinkage pattern	HR^+^/HER2^−^				HER2^+^				TNBC			
	N (151)	pCR (n = 15)	Non-pCR (n = 136)	*p*	N (123)	pCR (n = 68)	Non-pCR (n = 55)	*p*	N (71)	pCR (n = 30)	Non-pCR (n = 41)	*p*
CS	78 (52%)	4 (27%)	74 (54%)	0.167	67 (54%)	40 (59%)	27 (49%)	0.167	45 (63%)	25 (83%)	20 (49%)	NA
CS to small foci	1 (0.7%)	1 (6.7%)	0 (0%)	(CS vs. DIO)	9 (7.3%)	9 (13%)	0 (0%)	(CS vs. DIO)	3 (4.2%)	3 (10%)	0 (0%)	(CS vs. DIO)
Simple CS	74 (49%)	3 (20%)	71 (52%)	0.050	50 (41%)	27 (40%)	23 (42%)	< 0.001^a^	40 (56%)	21 (70%)	19 (46%)	< 0.001^a^
CS plus decreased enhancement	3 (2.0%)	0 (0%)	3 (2.2%)	(CS vs. SD)	8 (6.5%)	4 (5.9%)	4 (7.3%)	(CS vs. SD)	2 (2.8%)	1 (3.3%)	1 (2.4%)	(CS vs. SD)
DD	29 (19%)	11 (73%)	18 (13%)	< 0.001 ^a^	41 (33%)	25 (37%)	16 (29%)	0.167	13 (18%)	5 (17%)	8 (20%)	0.059
CS with surrounding lesions	7 (4.6%)	3 (20%)	4 (2.9%)	(DD vs. CS)	9 (7.3%)	4 (5.9%)	5 (9.1%)	(DD vs. CS)	5 (7.0%)	2 (6.7%)	3 (7.3%)	(DD vs. CS)
Residual multinodular lesions	22 (15%)	8 (53%)	14 (10%)	< 0.001 ^a^ (DD vs. SD)	32 (26%)	21 (31%)	11 (20%)	< 0.001^a^ (DD vs. SD)	8 (11%)	3 (10%)	5 (12%)	0.007^a^ (DD vs. SD)
DIO	3 (2.0%)	0 (0%)	3 (2.2%)	0.089 (DD vs. DIO)	3 (2.4%)	2 (2.9%)	1 (1.8%)	0.167 (DD vs. DIO)	0 (0%)	0 (0%)	0 (0%)	NA (DD vs. DIO)
SD	41 (27%)	0 (0%)	41 (30%)	NA (SD vs. DIO)	12 (10%)	1 (1.5%)	11 (20%)	0.014 (SD vs. DIO)	13 (18%)	0 (0%)	13 (32%)	NA (SD vs. DIO)

*pCR* pathologic complete response, *HER2* human epidermal growth factor receptor 2, *TNBC* triple-negative breast cancer, *HR* hormone receptor, *CS* concentric shrinkage, *DD* diffuse decrease, *DIO* decrease of intensity only, *SD* stable disease, *NA* not applicable

^a^Following adjustment for multiple comparisons with Bonferroni’s correction, the statistically significant *p* values were annotated (*p* < 0.00833)

After early NAT, the DD pattern had the highest pCR rate (11/29 [38%]) in HR^+^/HER2^−^ subtype compared with the CS pattern (4/78 [5.1%], *p* < 0.001) and no pCR case was found in the DIO (0/3) and SD (0/41) patterns. Considering the low pCR rate in HR^+^/HER2^−^ subtype, we subsequently investigated the correlation between TSP and pNR (Additional file 1: Table S5). The HR^+^/HER2^−^ subtype presenting with the DD pattern (24/29 [83%]) after early NAT had the highest non-pNR rate compared with the CS pattern (48/78 [62%], *p* = 0.006) and SD pattern (17/41 [41%], *p* < 0.001), and no pNR case was found in the DIO (0/3) pattern. The CS (40/67 [60%]), DD (25/41 [61%]) and DIO (2/3 [67%]) patterns had the considerable pCR rate in HER2^+^ subtype. The CS pattern (25/45 [56%]) had the highest pCR rate, followed by the DD pattern (5/13 [38%]) in TNBC. Especially for CS to small foci pattern, 100% pCR rate was obtained after NAT in each subtype despite the low incidence rate (HR^+^/HER2^−^: 1/1, HER2^+^: 9/9, and TNBC: 3/3). The SD pattern had the highest non-pCR rate in each molecular subtype as 41/41 (100%) for HR^+^/HER2^−^ (SD vs. DD, *p* < 0.001), as 11/12 (92%) for HER2^+^ (SD vs. CS, *p* < 0.001; SD vs. DD, *p* < 0.001), as 13/13 (100%) for TNBC (SD vs. CS, *p* < 0.001; SD vs. DD, *p* = 0.007) (Table 3).

**Fig. 4:**
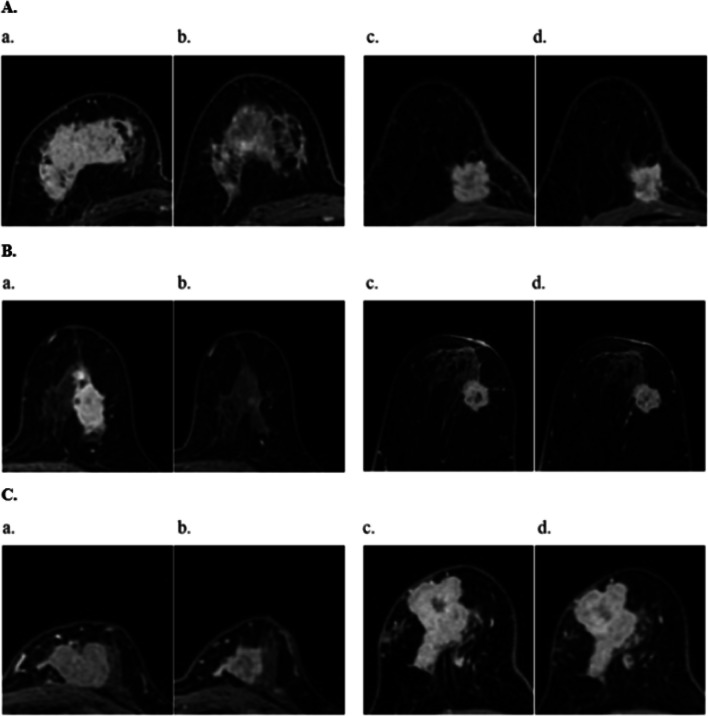
**A** Invasive ductal carcinoma (HR^+^/HER2^−^) with pathologic complete response (pCR) after NAT in a 49-year-old woman: (a) pretreatment: a 71 mm mass occupying most glands in the upper right quadrant; (b) early neoadjuvant therapy (NAT): the lesions showed shrinkage with residual multinodular lesions (DD pattern). Invasive ductal carcinoma (HR^+^/HER2^−^) with non-pCR after NAT in a 70-year-old woman: (c) pretreatment: a 25 mm mass in the upper right quadrant; (d) early NAT: the lesions showed the simple concentric shrinkage (CS pattern) with a diameter reduction of 4 mm. **B** Invasive ductal carcinoma (HER2^+^) with pCR after NAT in a 44-year-old woman: (a) pretreatment: a 45 mm mass in the upper left quadrant; (b) early NAT: the lesion size was notably diminished with only residual enhancement foci (CS: CS to small foci pattern). Invasive ductal carcinoma (HER2^+^) with non-pCR after NAT in a 58-year-old woman: (c) pretreatment: a 29 mm mass in the upper left quadrant; (d) early NAT: the lesions showed the stable disease (SD pattern) with no changes in size or morphology. **C** Invasive ductal carcinoma (TNBC) with pCR after NAT in a 53-year-old woman: (a) pretreatment: a 43 mm mass in the upper left quadrant; (b) early NAT: the lesions showed the simple concentric shrinkage (CS pattern) with a diameter reduction of 13 mm. Invasive ductal carcinoma (TNBC) with non-pCR after NAT in a 65-year-old woman: (c) pretreatment: a 50 mm mass in the upper right quadrant; (d) early NAT: the lesions showed the stable disease (SD pattern) with no changes in size or morphology

Multivariate analysis showed that early DD pattern (OR = 12.08; 95% CI 3.34–43.75; *p* < 0.001) predicted pCR independently of the change in tumor size (OR = 1.37; 95% CI 0.94–2.01; *p* = 0.106) (Table 4), and early DD pattern (OR = 0.29; 95% CI 0.10–0.88; *p* = 0.029) emerged as an independent predictor of pNR in addition to the change in tumor size (OR = 0.65; 95% CI 0.45–0.95; *p* = 0.027) in HR^+^/HER2^−^ subtype (Additional file 1: Table S6). In HER2^+^ subtype, univariate analysis showed that early SD pattern and the change in tumor size were associated with pCR; multivariate analysis showed that the change in tumor size (OR = 1.61; 95% CI 1.22–2.13; *p* = 0.001) was the only independent factor to predict pCR. In TNBC, univariate analysis showed that early change in tumor size (OR = 1.61; 95% CI 1.22–2.11, *p* = 0.001) was the only factor to predict pCR (Fig. 4). Compared with the change in tumor size, the SD pattern achieved a higher NPV in HER2^+^ and TNBC (Additional file 1: Table S7).

**Table 4:** Univariate and multivariate analysis of factors associated with pCR according to different molecular subtypes in the primary analysis cohort

Characteristics	Univariate analysis			Multivariate analysis		
	OR	95% CI	*p*	OR	95% CI	*p*
*HR*^*+*^*/HER2*^*−*^						
Tumor size (mm)						
D_pre_^a^	1.00	0.97–1.03	0.786	-	-	-
D_early_^a^	0.98	0.94–1.02	0.259	-	-	-
∆D_early_%^b^	1.59	1.16–2.19	0.004	1.37	0.94–2.01	0.106
Shrinkage pattern						
CS	Ref	Ref	Ref	Ref	Ref	Ref
DD	11.31	3.22–39.66	< 0.001	12.08	3.34–43.75	< 0.001
DIO	NA	NA	NA	NA	NA	NA
SD	NA	NA	NA	NA	NA	NA
*HER2*^*+*^						
Tumor size (mm)						
D_pre_^a^	0.98	0.97–1.00	0.115	–	–	–
D_early_^a^	0.96	0.93–0.98	0.001	–	–	–
∆D_early_%^b^	1.66	1.31–2.11	< 0.001	1.61	1.22–2.13	0.001
Shrinkage pattern						
CS	Ref	Ref	Ref	Ref	Ref	Ref
DD	1.06	0.48–2.34	0.896	1.04	0.45–2.44	0.921
DIO	1.35	0.12–15.64	0.810	6.14	0.42–83.23	0.173
SD	0.06	0.01–0.50	0.009	0.25	0.03–2.35	0.224
*TNBC*						
Tumor size (mm)						
D_pre_^a^	0.99	0.97–1.01	0.258	–	–	–
D_early_^a^	0.96	0.93–1.00	0.024	–	–	–
∆D_early_%^b^	1.61	1.22–2.11	0.001	–	–	–
Shrinkage pattern						
CS	Ref	Ref	Ref	–	–	–
DD	0.50	0.14–1.77	0.282	–	–	–
DIO	NA	NA	NA	–	–	–
SD	NA	NA	NA	–	–	–

*pCR* pathologic complete response, *NAT* neoadjuvant therapy, *HER2* human epidermal growth factor receptor 2, *TNBC* triple-negative breast cancer, *HR* hormone receptor, *CS* concentric shrinkage, *DD* diffuse decrease, *DIO* decrease of intensity only, *SD* stable disease, *OR* odds ratio, *Ref* reference, *NA* not applicable, *D*_*pre*_ the tumor size at Pre-MRI, *D*_*early*_ the tumor size after early NAT, *∆D*_*early*_*%* the percentage changes in tumor size after early NAT (continuous variable for 10% increment)

^a^D_pre_ and D_early_ were analyzed only in univariate analysis

^b^∆Dearly% with higher OR was used in multivariate analysis; NA was due to the fact that the shrinkage pattern had zero samples in either the pCR or non-pCR group

### The 1st-timepoint Subgroup Analysis

Additional file 1: Table S8 shows the distribution of TSP for each subtype in the subgroup analysis cohorts. At 1st-timepoint, the CS pattern had the highest frequency in each molecular subtype (45/100 [45%] for HR^+^/HER2^−^, 56/94 [60%] for HER2^+^ and 32/51 [63%] for TNBC), with mainly simple CS pattern. The DD pattern had 13/100 (13%) in HR^+^/HER2^−^, 23/94 (24%) in HER2^+^ and 5/51 (10%) in TNBC. The SD pattern had 40/100 (40%) in HR^+^/HER2^−^, 12/94 (13%) in HER2^+^ and 14/51 (27%) in TNBC. The DIO pattern rarely appeared at 1st-timepoint, with only 2/100 (2.0%) in HR^+^/HER2^−^, 3/94 (3.2%) in HER2^+^ and 0/51 in TNBC.

At 1st-timepoint, the DD pattern (5/13 [38%]) had the highest pCR rate in HR^+^/HER2^−^ subtype compared with the CS pattern (3/45 [6.7%], *p* = 0.002) and no pCR case was found in the DIO (0/2) and SD (0/40) patterns. All patients presenting with the DD pattern showed non-pNR (13/13 [100%]) in HR^+^/HER2^−^ subtype (Additional file 1: Table S5). The CS (36/56 [64%]) and DIO (2/3 [67%]) pattern had the considerable pCR rate in HER2^+^ subtype. The CS pattern (19/32 [59%]) and DD pattern (3/5 [60%]) had the considerable pCR rate in TNBC. The SD pattern had the highest non-pCR rate in each molecular subtype as 40/40 (100%) for HR^+^/HER2^−^ (SD vs. DD, *p* < 0.001), as 11/12 (92%) for HER2^+^ (SD vs. CS, *p* < 0.001; SD vs. DD, *p* = 0.002), as 13/14 (93%) for TNBC (SD vs. CS, *p* < 0.001; SD vs. DD, *p* = 0.006) (Additional file 1: Table S8).

In 1st-timepoint subgroup analysis, multivariate analysis showed that the DD pattern (OR = 9.99; 95% CI 1.78–56.04; *p* = 0.009) predicted pCR independently of the change in tumor size (OR = 0.87; 95% CI 0.46–1.64; *p* = 0.659) in HR^+^/HER2^−^ subtype. In HER2^+^ subtype, univariate analysis showed that the SD pattern and change in tumor size were associated with pCR; multivariate analysis showed that the change in tumor size was the only independent factor to predict pCR (OR = 1.75; 95% CI 1.20–2.56; *p* = 0.004). In TNBC, univariate analysis showed that the SD pattern (OR = 0.05; 95% CI 0.01–0.45, *p* = 0.007) and change in tumor size (OR = 1.94; 95% CI 1.29–2.92; *p* = 0.001) were associated with pCR, but the differences were not statistically significant in multivariate analysis (Additional file 1: Table S9). The result of complete 1st-timepoint analysis was consistent with the primary analysis.

### The 2nd-timepoint subgroup analysis

At 2nd-timepoint, the CS pattern had the highest frequency in each molecular subtype (50/94 [53%] for HR^+^/HER2^−^, 37/74 [50%] for HER2^+^ and 24/39 [62%] for TNBC), with mainly simple CS pattern. The DD pattern had 22/94 (23%) in HR^+^/HER2^−^, 30/74 (41%) in HER2^+^ and 11/39 (28%) in TNBC. The SD pattern had 20/94 (21%) in HR^+^/HER2^−^, 5/74 (6.8%) in HER2^+^ and 4/39 (10%) in TNBC. The DIO pattern rarely appeared after early NAT, with only 2/94 (2.1%) in HR^+^/HER2^−^, 2/74 (2.7%) in HER2^+^ and 0/39 in TNBC (Additional file 1: Table S8).

The DD pattern (6/22 [27%]) had the highest pCR rate in HR^+^/HER2^−^ subtype compared with the CS pattern (3/50 [6.0%], *p* = 0.003) and no pCR case was found in the DIO (0/2) and SD (0/20) patterns. Additionally, the DD pattern (17/22 [77%]) had the highest non-pNR rate compared with the CS pattern (32/50 [64%], *p* = 0.044) and SD pattern (6/20 [30%], *p* < 0.001) in HR^+^/HER2^−^ subtype (Additional file 1: Table S5). The CS (23/37 [62%]) and DD (20/30 [67%]) pattern had the considerable pCR rate in HER2^+^ subtype, while the CS pattern had the highest pCR rate in TNBC (12/24 [50%]). All patients presenting with the SD pattern showed non-pCR in each subtype (Additional file 1: Table S8).

In 2nd-timepoint subgroup analysis, multivariate analysis showed that the DD pattern (OR = 7.72; 95% CI 1.55–38.53; *p* = 0.013) emerged as an independent predictor of pCR in addition to the change in tumor size (OR = 1.61; 95% CI 1.01–2.59, *p* = 0.046) in HR^+^/HER2^−^ subtype. Univariate analysis showed that the change in tumor size was the only factor to predict pCR in HER2^+^ subtype (OR = 1.45; 95% CI 1.10–1.91; *p* = 0.008) and TNBC (OR = 1.43; 95% CI 1.03–1.98; *p* = 0.033) (Additional file 1: Table S9).

Compared with the primary cohort and complete 1st-timepoint subgroup analysis, the DD pattern was no longer the only independent pCR predictor in HR^+^/HER2^−^ subtype at 2nd-timepoint analysis (Additional file 1: Table S9). In HER2^+^ and TNBC, the change in tumor size at 1st-timepoint (HER2^+^ : OR = 1.86, AUC = 0.731, both *p* < 0.001; TNBC: OR = 1.94, *p* = 0.001; AUC = 0.804, *p* < 0.001) had a greater impact on pCR prediction than that at 2nd-timepoint (HER2^+^ : OR = 1.45, *p* = 0.008; AUC = 0.677, *p* = 0.007; TNBC: OR = 1.43, *p* = 0.033; AUC = 0.693, *p* = 0.034) (Additional file 1: Table S9, Fig. S1).

### Early imaging response strategy map

**Fig. 5:**
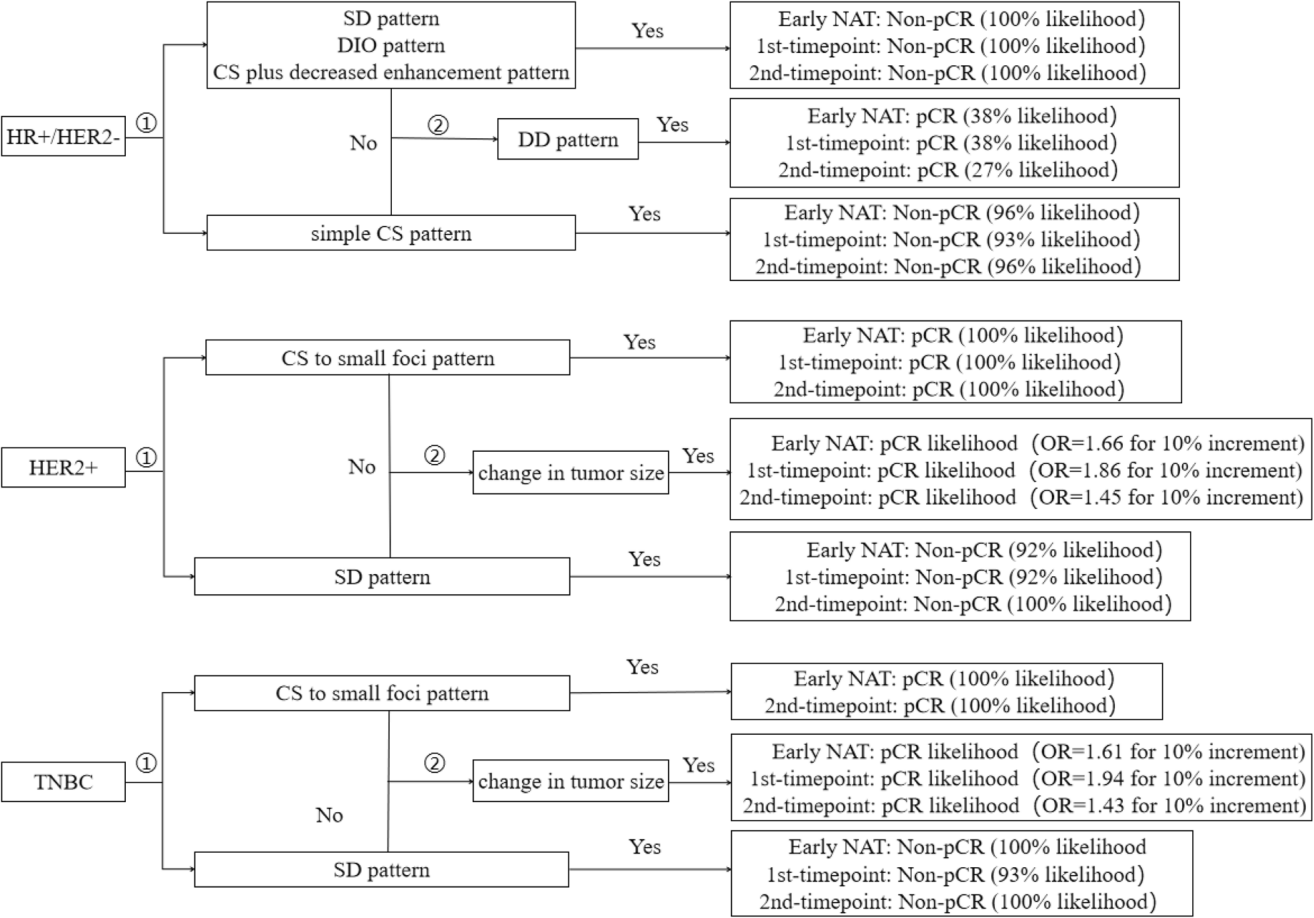
Strategy map for predicting pCR based on shrinkage patterns and the change in tumor size in each subtype

Strategy maps based on TSP and the change in tumor size in each subtype are plotted. For pCR prediction in HR^+^/HER2^−^ subtype, radiologists should first identify the non-pCR patients with the SD pattern (or a few DIO or CS plus decreased enhancement patterns) and the simple CS pattern; Then evaluate whether the patient has the DD pattern, which is a potential pCR manifestation although there is only 38% likelihood of pCR. In HER2^+^ and TNBC, we should first identify a pCR patient with the CS to small foci pattern or a non-pCR patient with the SD pattern; If neither, the likelihood of pCR depends on the tumor size change with OR of 1.86 in HER2^+^ and 1.94 in TNBC for 10% increment at 1st-timepoint, for example (Fig. 5).

**Fig. 6:**
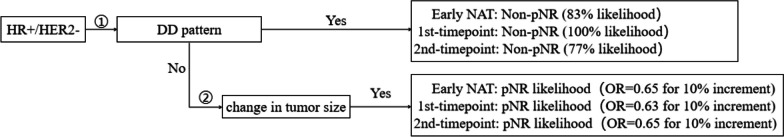
Strategy map for predicting pNR based on shrinkage patterns and the change in tumor size in HR^+^/HER2^−^ subtype

For pNR prediction in HR^+^/HER2^−^ subtype, radiologists should first identify whether the patient has the DD pattern, which is a highly likely non-pNR manifestation. If the patient does not have the DD pattern, the likelihood of pNR depends on the tumor size change with OR of 0.63 for 10% increment at 1st-timepoint, for example (Fig. 6).

## Discussion

In the modern era with updated neoadjuvant therapy regimens, the present study evaluated TSP on DCE-MRI after early NAT and its association with pCR within each breast cancer subtype. Our findings indicated that the DD pattern after early NAT, particularly at 1st-timepoint, was a tumor response marker independent of the size change in HR^+^/HER2^−^ subtype; the SD pattern in HER2^+^ and TNBC after early NAT strongly indicated non-pCR. TSP could serve as additional straightforward and comprehensible indicators of treatment response in addition to the change in tumor size.

The classification and definition of TSP at MRI have not been consistently recognized and unified. The CS or non-CS patterns after NAT and further refinement of non-CS pattern at mid-NAT were commonly used [11, 17, 20]. Based on Fukada et al.’s study [11], we developed four-category TSP and subdivided CS and DD pattern to suit early NAT response. The overall loss of cellularity after NAT was not always reflected by a decreased tumor size. NAT can cause different changes in the nucleus and cytoplasm of tumors, leading to changes in overall morphology and exhibiting different TSP [29]. Compared to HER2^+^ subtype, HR^+^/HER2^−^ subtype tends to grow slowly, showing low apoptosis rates and genetic instability [11]. The internal heterogeneity of these tumors causes them to shrink inconsistently and crumble into small foci or scattered cells. The sparse microvascular distribution in HR^+^/HER2^−^ subtype also leads to uneven drug delivery, which tends to have the DD pattern after NAT. In our study, the DD pattern after early NAT tended toward pCR in HR^+^/HER2^−^ subtype, mainly at 1st-timepoint, independent of size change. Reis et al. [20] reported that early fragmentation pattern after 2 months neoadjuvant endocrine therapy suggested effective treatment in ER^+^/HER2^−^ subtype. The DD pattern may be the early manifestation of HR^+^/HER2^−^ subtype response to NAT earlier than size reduction. Our study recommended introducing the TSP for early imaging response strategies in HR^+^/HER2^−^ subtype.

HER2^+^ and TNBC have the highest proportion of CS pattern, which is consistent with previous studies for mid- and post-NAT evaluation [30–33]. Animal studies [34] on tumor subregions have shown that tumor margins of HER2^+^ and TNBC are distributed with abundant microvessels and high cell proliferation. Abundant vessels facilitated the delivery of drugs thus making these tumors more sensitive to therapy, resulting in more homogeneous cell reduction and shrinkage. Heacock et al. [18] and Eom et al. [19] reported that the CS pattern was a stronger predictor of pCR in HER2^+^ and TNBC after NAT. Different from post-NAT timepoint, the CS pattern after early NAT did not show a significant pCR tendency compared with the DD pattern, but the SD pattern strongly indicated non-pCR in HER2^+^ and TNBC. The change in tumor size was still a strong predictor of pCR in HER2^+^ and TNBC after early NAT, even at 1st-timepoint.

Based on the observed TSP, we develop an early imaging response strategy for each subtype of breast cancer. By employing this strategy, clinicians can effectively inform patients of the potential pathological response and its associated probability. The results of 1st-timepoint subgroup analysis were consistent with those of the primary analysis cohort, indicating TSP can be evaluated even after the first cycle of NAT. This easily understandable approach can assist clinicians in modifying treatment plans to enhance effectiveness, minimize unnecessary adverse effects, and improve disease-free survival rates. However, noted that the signal intensities of DCE-MRI are influenced by imaging protocols and gadolinium-based contrast agents from different vendors, therefore TSP such as “DIO” and “CS plus decreased enhancement” may be susceptible to potential influences. To mitigate the variability in TSP evaluation after treatment, it is crucial to utilize uniformity MRI scanners, standardized contrast agents, and skilled radiologists in the serial imaging evaluation of the identical patient during NAT.

Our study had some limitations. First, despite the overall large sample size, the number of each subtype was limited. Enhancing the sample size for each subtype would augment the strength of our evidence. Secondly, the homogeneity of the study sample and the data acquisition method mitigated the influence of confounding variables, but the result may be specific to this acquisition technique. The performance of our findings on a different scanner platform, or with different imaging protocol is unknown. Finally, our study employed visual assessment conducted by radiologists, which was both qualitative and subjective. Future research should strive to incorporate artificial intelligence techniques to enable rapid, objective and reproducible analysis of TSP.

## Conclusion

The TSP after early NAT may serve as an additional straightforward and comprehensible indicator of treatment response in addition to the change in tumor size. Specifically, the diffuse decrease pattern in HR^+^/HER2^−^ subtype is a tumor response marker independent of the size change, and the stable disease in HER2^+^ and TNBC strongly indicates non-pCR at 1st-timepoint.

### Supplementary Information

**Additional file 1**. **Table S1**. Participants characteristics in the primary analysis cohort. **Table S2**. Participants characteristics in the subgroup analysis cohorts. **Table S3.** Inter-reader agreement for tumor shrinkage patterns in each cohort. **Table S4.** Inconsistent shrinkage pattern distribution between two readers. **Table S5.** MRI-based tumor shrinkage patterns association with pNR in HR^+^/HER2^−^ subtype. **Table S6.** Univariate and multivariate analysis of factors associated with pNR in HR^+^/HER2^−^ subtype. **Table S7**. The diagnostic efficacy of factors in each molecular subtype. **Table S8.** MRI-based tumor shrinkage patterns association with pCR according to different molecular subtypes in the subgroup analysis cohorts. **Table S9**. Univariate and multivariate analysis of factors associated with pCR according to different molecular subtypes in the subgroup analysis cohorts. **Figure S1.** Receiver operating characteristic (ROC) curves of the change in tumor size (continuous variable) at 1st-timepoint and 2nd-timepoint for pathologic complete response (pCR) prediction in the breast.

## Data Availability

The datasets used and analyzed during the current study are available from the corresponding author on reasonable request.

## Abbreviations

TSP: Tumor shrinkage patterns
CS: Concentric shrinkage
DD: Diffuse decrease
DIO: Decrease of intensity only
SD: Stable disease
HER2: Human epidermal growth factor receptor 2
HR: Hormone receptor
TNBC: Triple-negative breast cancer
NAT: Neoadjuvant therapy
pCR: Pathologic complete response
