# Supplementary material for: MRI-based tumor shrinkage patterns after early neoadjuvant therapy in breast cancer: correlation with molecular subtypes and pathological response after therapy
Source: Breast Cancer Res. 2024 Feb 12;26:26. doi: 10.1186/s13058-024-01781-1 (PMC10863121; doi:10.1186/s13058-024-01781-1)
Supplement: Supplementary file 1 — Additional file 1. Table S1. Participants characteristics in the primary analysis cohort. Table S2. Participants characteristics in the subgroup analysis cohorts. Table S3. Inter-reader agreement for tumor shrinkage patterns in each cohort. Table S4. Inconsistent shrinkage pattern distribution between two readers. Table S5. MRI-based tumor shrinkage patterns association with pNR in HR+/HER2− subtype. Table S6. Univariate and multivariate analysis of factors associated with pNR in HR+/HER2− subtype. Table S7. The diagnostic efficacy of factors in each molecular subtype. Table S8. MRI-based tumor shrinkage patterns association with pCR according to different molecular subtypes in the subgroup analysis cohorts. Table S9. Univariate and multivariate analysis of factors associated with pCR according to different molecular subtypes in the subgroup analysis cohorts. Figure S1. Receiver operating characteristic (ROC) curves of the change in tumor size (continuous variable) at 1st-timepoint and 2nd-timepoint for pathologic complete response (pCR) prediction in the breast. [file 13058_2024_1781_MOESM1_ESM.docx]

**Supplementary material**

**Table S1** Participants characteristics in the primary analysis cohort

| Characteristic | All patients  n=345* | pCR  (n=113) | Non-pCR  (n=232) | *p* |
| --- | --- | --- | --- | --- |
| Age (year), IQR | 51 (42,58) | 51 (43,56) | 52 (41,58) | 0.270 |
| Tumor size (mm), IQR |  |  |  |  |
| D_pre_ | 37 (27,49) | 35 (25,47) | 38 (28,51) | 0.101 |
| D_early_ | 27 (19,40) | 21 (15,32) | 30 (22,44) | < 0.001 |
| ΔD_early_% | 22 (10,37) | 36 (24,51) | 16 (4,28) | < 0.001 |
| Menopausal status |  |  |  | 0.817 |
| Premenopausal | 174 (50) | 58 (51) | 116 (50) |  |
| Postmenopausal | 171 (50) | 55 (49) | 116 (50) |  |
| TNM |  |  |  | 0.186 |
| ⅡA | 64 (19) | 27 (24) | 37 (16) |  |
| ⅡB | 127 (37) | 43 (38) | 84 (36) |  |
| ⅢA | 50 (15) | 13 (12) | 37 (16) |  |
| ⅢB | 27 (7.8) | 5 (4.4) | 22 (10) |  |
| ⅢC | 77 (22) | 25 (22) | 52 (22) |  |
| Histologic grade |  |  |  | < 0.001 |
| 2 | 239 (69) | 63 (56) | 176 (76) |  |
| 3 | 106 (31) | 50 (44) | 56 (24) |  |
| Molecular subtype |  |  |  | < 0.001 |
| HR+/HER2- | 151 (44) | 15 (13) | 136 (59) |  |
| HER2+ | 123 (36) | 68 (60) | 55 (24) |  |
| TNBC | 71 (21) | 30 (27) | 41 (18) |  |
| NAT regimen |  |  |  | < 0.001 |
| Anthracycline-based | 29 (8.4) | 2 (1.8) | 27 (12) |  |
| Taxane-based | 123 (36) | 67 (59) | 56 (24) |  |
| Anthracycline and taxane-based | 193 (56) | 44 (39) | 149 (64) |  |
| FGT |  |  |  | 0.433 |
| Scattered | 70 (20) | 19 (17) | 51 (22) |  |
| Heterogeneously | 211 (61) | 70 (62) | 141 (61) |  |
| Extremely dense | 64 (19) | 24 (21) | 40 (17) |  |
| BPE |  |  |  | 0.606 |
| Minimal | 35 (10) | 9 (8.0) | 26 (11) |  |
| Mild | 192 (56) | 64 (57) | 128 (55) |  |
| Moderate | 88 (26) | 32 (28) | 56 (24) |  |
| Marked | 30 (8.7) | 8 (7) | 22 (10) |  |
| Enhancement type |  |  |  | 0.382 |
| Mass | 281 (81) | 95 (84) | 186 (80) |  |
| Non-mass | 64 (19) | 18 (16) | 46 (20) |  |
| Multiplicity |  |  |  | 0.593 |
| Single lesion | 129 (37) | 40 (35) | 89 (38) |  |
| Multi-lesion | 216 (63) | 73 (65) | 143 (62) |  |
| Shape |  |  |  | 0.099 |
| Round or oval | 68 (20) | 28 (25) | 40 (17) |  |
| Irregular | 277 (80) | 85 (75) | 192 (83) |  |
| Margin |  |  |  | 0.698 |
| Circumscribed | 24 (7.0) | 7 (6.2) | 17 (7.3) |  |
| Not circumscribed | 321 (93) | 106 (94) | 215 (93) |  |
| Kinetics |  |  |  | 0.582 |
| Persistent | 8 (2.3) | 3 (2.7) | 5 (2.2) |  |
| Plateau | 123 (36) | 36 (32) | 87 (38) |  |
| Washout | 214 (62) | 74 (66) | 140 (60) |  |

Unless otherwise specified, data are numbers of participants, with percentages in parentheses

pCR pathologic complete response, HER2 human epidermal growth factor receptor 2, TNBC triple-negative breast cancer, HR hormone receptor, NAT neoadjuvant therapy, BPE background parenchymal enhancement, FGT fibroglandular tissue, D_pre_ the tumor size at Pre-MRI, D_early_ the tumor size after early NAT, ∆D_early_% the percentage changes in tumor size after early NAT (continuous variable)

*345 MRI including 138 1st-MRI and 207 2nd-MRI.

**Table S2** Participants characteristics in the subgroup analysis cohorts

| Characteristics | 1st-timepoint Subgroup | | | | 2nd-timepoint Subgroup | | | |
| --- | --- | --- | --- | --- | --- | --- | --- | --- |
|  | All patients  (n=245) | pCR  (n=82) | Non-pCR  (n=163) | *p* | All patients  (n=207) | pCR  (n=69) | Non-pCR  (n=138) | *p* |
| Age (year), IQR | 51 (42,58) | 51 (42,56) | 52 (42,59) | 0.110 | 50 (41,58) | 51 (41,57) | 50 (41,58) | 0.798 |
| Tumor size (mm), IQR |  |  |  |  |  |  |  |  |
| D_pre_ | 36 (27,49) | 35 (26,47) | 36 (28,50) | 0.328 | 37 (28,49) | 35 (25,48) | 38 (28,49) | 0.143 |
| D_1st/2nd_ | 29 (21,43) | 25 (17,35) | 31 (23,45) | <0.001 | 26 (18,37) | 19 (13,27) | 29 (21,40) | < 0.001 |
| ΔD_1st_/ΔD_2nd_% | 16 (5,29) | 31 (16,40) | 11 (2,21) | < 0.001 | 26 (13,40) | 38 (26,54) | 19 (6,34) | < 0.001 |
| Menopausal status |  |  |  | 0.300 |  |  |  | 0.768 |
| Premenopausal | 129 (53) | 47 (57) | 82 (50) |  | 105 (51) | 34 (49) | 71 (51) |  |
| Postmenopausal | 116 (47) | 35 (43) | 81 (50) |  | 102 (49) | 35 (51) | 67 (49) |  |
| TNM |  |  |  | 0.374 |  |  |  | 0.185 |
| ⅡA | 49 (20) | 21 (26) | 28 (17) |  | 33 (16) | 14 (20) | 19 (14) |  |
| ⅡB | 79 (32) | 27 (33) | 52 (32) |  | 84 (41) | 29 (42) | 55 (40) |  |
| ⅢA | 33 (14) | 9 (11) | 24 (15) |  | 31 (15) | 8 (12) | 23 (17) |  |
| ⅢB | 24 (10) | 5 (6.1) | 19 (12) |  | 13 (6.3) | 1 (1.4) | 12 (8.7) |  |
| ⅢC | 60 (25) | 20 (24) | 40 (25) |  | 46 (22) | 17 (25) | 29 (21) |  |
| Histologic grade |  |  |  | 0.003 |  |  |  | 0.021 |
| 2 | 165 (67) | 45 (55) | 120 (74) |  | 150 (73) | 43 (62) | 107 (78) |  |
| 3 | 80 (33) | 37 (45) | 43 (26) |  | 57 (28) | 26 (38) | 31 (23) |  |
| Molecular subtype |  |  |  | < 0.001 |  |  |  | < 0.001 |
| HR+/HER2- | 100 (41) | 8 (10) | 92 (56) |  | 94 (45) | 9 (13) | 85 (62) |  |
| HER2+ | 94 (38) | 51 (62) | 43 (26) |  | 74 (36) | 44 (64) | 30 (22) |  |
| TNBC | 51 (21) | 23 (28) | 28 (17) |  | 39 (19) | 16 (23) | 23 (17) |  |
| NAT regimen |  |  |  | < 0.001 |  |  |  | < 0.001 |
| Anthracycline-based | 17 (6.9) | 2 (2.4) | 15 (9.2) |  | 16 (7.7) | 0 (0) | 16 (12) |  |
| Taxane-based | 92 (38) | 49 (60) | 43 (26) |  | 76 (37) | 44 (64) | 32 (23) |  |
| Anthracycline and taxane-based | 136 (56) | 31 (38) | 105 (64) |  | 115 (56) | 25 (36) | 90 (65) |  |
| FGT |  |  |  | 0.250 |  |  |  | 0.920 |
| Scattered | 51 (21) | 15 (18) | 36 (22) |  | 39 (19) | 12 (17) | 27 (20) |  |
| Heterogeneously | 143 (58) | 45 (55) | 98 (60) |  | 125 (60) | 42 (61) | 83 (60) |  |
| Extremely dense | 51 (21) | 22 (27) | 29 (18) |  | 43 (21) | 15 (22) | 28 (20) |  |
| BPE |  |  |  | 0.670 |  |  |  | 0.285 |
| Minimal | 29 (12) | 8 (10) | 21 (13) |  | 14 (6.8) | 2 (2.9) | 12 (8.7) |  |
| Mild | 139 (57) | 45 (55) | 94 (58) |  | 113 (55) | 42 (61) | 71 (51) |  |
| Moderate | 58 (24) | 23 (28) | 35 (22) |  | 56 (27) | 19 (28) | 37 (27) |  |
| Marked | 19 (7.8) | 6 (7.3) | 13 (8.0) |  | 24 (12) | 6 (9) | 18 (13) |  |
| Enhancement type |  |  |  | 0.395 |  |  |  | 0.324 |
| Mass | 202 (82) | 70 (85) | 132 (81) |  | 166 (80) | 58 (84) | 108 (78) |  |
| Non-mass | 43 (18) | 12 (15) | 31 (19) |  | 41 (20) | 11 (16) | 30 (22) |  |
| Multiplicity |  |  |  | 0.956 |  |  |  | 1.000 |
| Single lesion | 98 (40) | 33 (40) | 65 (40) |  | 66 (32) | 22 (32) | 44 (32) |  |
| Multi-lesion | 147 (60) | 49 (60) | 98 (60) |  | 141 (68) | 47 (68) | 94 (68) |  |
| Shape |  |  |  | 0.200 |  |  |  | 0.521 |
| Round or oval | 54 (22) | 22 (27) | 32 (20) |  | 37 (18) | 14 (20) | 23 (17) |  |
| Irregular | 191 (78) | 60 (73) | 131 (80) |  | 170 (82) | 55 (80) | 115 (83) |  |
| Margin |  |  |  | 0.991 |  |  |  | 0.462 |
| Circumscribed | 15 (6.1) | 5 (6.1) | 10 (6.1) |  | 16 (7.7) | 4 (5.8) | 12 (8.7) |  |
| Not circumscribed | 230 (94) | 77 (94) | 153 (94) |  | 191 (92) | 65 (94) | 126 (91) |  |
| Kinetics |  |  |  | 0.210 |  |  |  | 0.321 |
| Persistent | 5 (2.0) | 3 (3.7) | 2 (1.2) |  | 4 (1.9) | 1 (1.4) | 3 (2.2) |  |
| Plateau | 86 (35) | 24 (29) | 62 (38) |  | 71 (34) | 19 (28) | 52 (38) |  |
| Washout | 154 (63) | 55 (67) | 99 (61) |  | 132 (64) | 49 (71) | 83 (60) |  |

Unless otherwise specified, data are numbers of participants, with percentages in parentheses

pCR pathologic complete response, HER2 human epidermal growth factor receptor 2, TNBC triple-negative breast cancer, HR hormone receptor, NAT neoadjuvant therapy, BPE background parenchymal enhancement, FGT fibroglandular tissue, D_pre_ the tumor size at Pre-MRI, D_1st_ the tumor size at 1st-MRI, D_2nd_ the tumor size at 2nd-MRI, ∆D_1st_/∆D_2nd_% the percentage changes in tumor size at 1st-MRI or 2nd-MRI (continuous variable)

**Table S3** Inter-reader agreement for tumor shrinkage patterns in each cohort

| Shrinkage pattern | N | Consistency | Inconsistency | Inter-reader agreement | |
| --- | --- | --- | --- | --- | --- |
|  |  |  |  | Kappa (κ) | 95% CI |
| All evaluations | 452* | 410/452 | 42/452 | 0.917 | 0.891-0.943 |
| Primary analysis cohort | 345 | 317/345 | 28/345 | 0.929 | 0.902-0.956 |
| Subgroup analysis cohort | 245(1st -MRI) | 223/245 | 22/245 | 0.942 | 0.911-0.973 |
|  | 207 (2nd-MRI) | 187/207 | 20/207 | 0.941 | 0.907-0.975 |

*452 MRI including 245 1st-MRI and 207 2nd-MRI, of which 107 had both evaluable 1st -MRI and 207 2nd-MRI

**Table S4** Inconsistent shrinkage pattern distribution between two readers

| Patients | **1st -MRI** | | **2nd-MRI** | |
| --- | --- | --- | --- | --- |
|  | Reader 1 | Reader 2 | Reader 1 | Reader 2 |
| 1 | CS plus decreased enhancement | residual multinodular lesions* | residual multinodular lesions* | simple CS |
| 2 | simple CS* | residual multinodular lesions | CS plus decreased enhancement | CS to small foci* |
| 3 | residual multinodular lesions* | simple CS | CS plus decreased enhancement | residual multinodular lesions* |
| 4 | residual multinodular lesions | CS to small foci* | CS with surrounding lesions* | residual multinodular lesions |
| 5 | CS with surrounding lesions | simple CS* | residual multinodular lesions | simple CS* |
| 6 | CS with surrounding lesions* | CS plus decreased enhancement | CS with surrounding lesions* | simple CS |
| 7 | CS to small foci* | CS plus decreased enhancement | CS with surrounding lesions* | CS plus decreased enhancement |
| 8 | CS plus decreased enhancement | residual multinodular lesions* | CS with surrounding lesions | residual multinodular lesions* |
| 9 | CS with surrounding lesions* | residual multinodular lesions | simple CS* | CS with surrounding lesions |
| 10 | CS with surrounding lesions* | simple CS | residual multinodular lesions* | simple CS |
| 11 | simple CS | CS with surrounding lesions* | simple CS* | CS plus decreased enhancement |
| 12 | residual multinodular lesions* | simple CS | CS plus decreased enhancement | residual multinodular lesions* |
| 13 | CS plus decreased enhancement* | residual multinodular lesions | CS plus decreased enhancement* | residual multinodular lesions |
| 14 | CS plus decreased enhancement | residual multinodular lesions* | CS plus decreased enhancement | CS to small foci* |
| 15 | CS plus decreased enhancement | residual multinodular lesions* | CS plus decreased enhancement | residual multinodular lesions* |
| 16 | CS plus decreased enhancement* | simple CS | simple CS* | CS plus decreased enhancement |
| 17 | CS to small foci* | CS plus decreased enhancement | CS with surrounding lesions* | CS plus decreased enhancement |
| 18 | residual multinodular lesions* | CS plus decreased enhancement | CS with surrounding lesions* | simple CS |
| 19 | CS plus decreased enhancement | residual multinodular lesions* | CS plus decreased enhancement | CS to small foci* |
| 20 | CS plus decreased enhancement | residual multinodular lesions* | residual multinodular lesions* | CS plus decreased enhancement |
| 21 | CS with surrounding lesions* | simple CS |  |  |
| 22 | residual multinodular lesions* | CS plus decreased enhancement |  |  |

***** The ultimate shrinkage pattern was established through consensus between two readers or involving a third reader if necessary

**Table S5** MRI-based tumor shrinkage patterns association with pNR in HR+/HER2- subtype

| shrinkage pattern | Primary Analysis Cohort | | | | Subgroup Analysis Cohort | | | | | | | |
| --- | --- | --- | --- | --- | --- | --- | --- | --- | --- | --- | --- | --- |
|  |  |  |  |  | 1st-timepoint Subgroup | | | | 2nd-timepoint Subgroup | | | |
|  | N  (151) | pNR  (n=59) | non-pNR  (n=92) | *p* | N  (100) | pNR  (n=38) | non-pNR  (n=62) | *p* | N  (94) | pNR  (n=37) | non-pNR  (n=57) | *p* |
| CS | 78 (52%) | 30 (51%) | 48 (52%) | 0.049  (CS vs. DIO)  0.006*  (CS vs. SD) | 45 (45%) | 20 (53%) | 25 (40%) | 0.083  (CS vs. DIO)  0.160  (CS vs. SD) | 50 (53%) | 18 (49%) | 32 (56%) | 0.090  (CS vs. DIO)  0.002*  (CS vs. SD) |
| CS to small foci | 1 (0.7%) | 0 (0%) | 1 (1.1%) |  | 0 (0%) | 0 (0%) | 0 (0%) |  | 1 (1.1%) | 0 (0%) | 1 (1.8%) |  |
| simple CS | 74 (49%) | 29 (49%) | 45 (49%) |  | 43 (43%) | 19 (50%) | 24 (39%) |  | 48 (51%) | 18 (49%) | 30 (53%) |  |
| CS plus decreased enhancement | 3 (2.0%) | 1 (1.7%) | 2 (2.2%) |  | 2 (2.0%) | 1 (2.6%) | 1 (1.6%) |  | 1 (1.1%) | 0 (0%) | 1 (1.8%) |  |
| DD | 29 (19%) | 5 (8%) | 24 (26%) | 0.006*  (DD vs. CS)  <0.001*  (DD vs. SD) | 13 (13%) | 0 (0%) | 13 (21%) | <0.001*  (DD vs. CS)  <0.001*  (DD vs. SD) | 22 (23%) | 5 (14%) | 17 (30%) | 0.044  (DD vs. CS)  <0.001*  (DD vs. SD) |
| CS with surrounding lesions | 7 (4.6%) | 0 (0%) | 7 (7.6%) |  | 4 (4.0%) | 0 (0%) | 4 (6.5%) |  | 5 (5.3%) | 0 (0%) | 5 (8.8%) |  |
| residual multinodular lesions | 22 (15%) | 5 (8.5%) | 17 (18%) |  | 9 (9.0%) | 0 (0%) | 9 (15%) |  | 17 (18%) | 5 (14%) | 12 (21%) |  |
| DIO | 3 (2.0%) | 0 (0%) | 3 (3.3%) | 0.167  (DD vs. DIO) | 2 (2.0%) | 0 (0%) | 2 (3.2%) | NA  (DD vs. DIO) | 2 (2.1%) | 0 (0%) | 2 (3.5%) | 0.167  (DD vs. DIO) |
| SD | 41 (27%) | 24 (41%) | 17 (18%) | 0.014  (SD vs. DIO) | 40 (40%) | 18 (47%) | 22 (35%) | 0.083  (SD vs. DIO) | 20 (21%) | 14 (38%) | 6 (11%) | 0.020  (SD vs. DIO) |

pNR nonresponse group, HER2 human epidermal growth factor receptor 2, HR hormone receptor, CS concentric shrinkage, DD diffuse decrease, DIO decrease of intensity only, SD stable disease, NA not applicable

* Following adjustment for multiple comparisons with Bonferroni’s correction, the statistically significant *p* values were annotated (*p* < 0.00833)

**Table S6** Univariate and multivariate analysis of factors associated with pNR in HR+/HER2- subtype

| Characteristics | Univariate analysis | | | Multivariate analysis | | |
| --- | --- | --- | --- | --- | --- | --- |
|  | OR | 95%CI | *p* | OR | 95%CI | *p* |
| **Primary Analysis cohort** |  |  |  |  |  |  |
| Tumor size (mm) |  |  |  |  |  |  |
| D_pre_* | 1.00 | 0.98-1.02 | 0.884 | - | - | - |
| D_early_* | 1.01 | 0.99-1.03 | 0.327 | - | - | - |
| ∆D_early_%^†^ | 0.65 | 0.50-0.85 | 0.002 | 0.65 | 0.45-0.95 | 0.027 |
| Shrinkage pattern |  |  |  |  |  |  |
| CS | Ref | Ref | Ref | Ref | Ref | Ref |
| DD | 0.33 | 0.12-0.97 | 0.043 | 0.29 | 0.10-0.88 | 0.029 |
| DIO | NA | NA | NA | NA | NA | NA |
| SD | 2.26 | 1.05-4.88 | 0.038 | 0.97 | 0.34-2.79 | 0.961 |
| **1st-timepoint Subgroup** |  |  |  |  |  |  |
| Tumor size (mm) |  |  |  |  |  |  |
| D_pre_* | 0.99 | 0.97-1.01 | 0.482 | - | - | - |
| D_1st_* | 1.00 | 0.98-1.02 | 0.940 | - | - | - |
| ∆D_1st_%^†^ | 0.63 | 0.42-0.94 | 0.023 | 0.52 | 0.26-1.02 | 0.055 |
| Shrinkage pattern |  |  |  |  |  |  |
| CS | Ref | Ref | Ref | Ref | Ref | Ref |
| DD | NA | NA | NA | NA | NA | NA |
| DIO | NA | NA | NA | NA | NA | NA |
| SD | 1.02 | 0.43-2.41 | 0.959 | 0.39 | 0.11-1.43 | 0.156 |
| **2nd-timepoint Subgroup** |  |  |  |  |  |  |
| Tumor size (mm) |  |  |  |  |  |  |
| D_pre_* | 1.01 | 0.99-1.03 | 0.395 | - | - | - |
| D_2nd_* | 1.02 | 0.99-1.04 | 0.113 | - | - | - |
| ∆D_2nd_%^†^ | 0.65 | 0.47-0.90 | 0.009 | 0.71 | 0.46-1.09 | 0.117 |
| Shrinkage pattern |  |  |  |  |  |  |
| CS | Ref | Ref | Ref | Ref | Ref | Ref |
| DD | 0.52 | 0.17-1.66 | 0.270 | 0.43 | 0.13-1.45 | 0.173 |
| DIO | NA | NA | NA | NA | NA | NA |
| SD | 4.42 | 1.36-12.68 | 0.013 | 1.93 | 0.45-8.21 | 0.376 |

pNR nonresponse group, HER2 human epidermal growth factor receptor 2, HR hormone receptor, CS concentric shrinkage, DD diffuse decrease, DIO decrease of intensity only, SD stable disease, OR odds ratio, Ref reference, NA not applicable, D_pre_ the tumor size at Pre-MRI, D_early_ the tumor size after early NAT, D_1st_ the tumor size at 1st-MRI, D_2nd_ the tumor size at 2nd-MRI, ∆D_early_% the percentage changes in tumor size after early NAT (continuous variable for 10% increment), ΔD_1st_% the percentage changes in tumor size at 1st-MRI (continuous variable for 10% increment), ΔD_2nd_ % the percentage changes in tumor size at 2nd-MRI (continuous variable for 10% increment)

*D_pre_ and D_early_ were analyzed only in univariate analysis; ^†^∆Dearly% with higher OR was used in multivariate analysis; NA was due to the fact that the shrinkage pattern had zero samples in either the pNR or non-pNR group.

**Table S7** The diagnostic efficacy of factors in each molecular subtype

|  | Sensitivity | Specificity | Positive predictive value | Negative predictive value | Accuracy |
| --- | --- | --- | --- | --- | --- |
| **Primary Analysis Cohort** |  |  |  |  |  |
| HR+/HER2- |  |  |  |  |  |
| ΔD_early_%^†^ | 0.733 | 0.691 | 0.208 | 0.959 | 0.695 |
| TSP (non-SD vs SD) ^†^ | 1.000 | 0.302 | 0.136 | 1.000 | 0.371 |
| HER2+ |  |  |  |  |  |
| ΔD_early_%^†^ | 0.868 | 0.582 | 0.720 | 0.781 | 0.740 |
| TSP (non-SD vs SD) ^†^ | 0.985 | 0.200 | 0.604 | 0.917 | 0.634 |
| TNBC |  |  |  |  |  |
| ΔD_early_%^†^ | 0.767 | 0.683 | 0.639 | 0.800 | 0.718 |
| TSP (non-SD vs SD) ^†^ | 1.000 | 0.317 | 0.517 | 1.000 | 0.606 |
| **1st-timepoint Subgroup** |  |  |  |  |  |
| HR+/HER2- |  |  |  |  |  |
| ΔD_1st_%^†^ | 0.625 | 0.826 | 0.238 | 0.962 | 0.810 |
| TSP (non-SD vs SD) ^†^ | 1.000 | 0.435 | 0.133 | 1.000 | 0.480 |
| HER2+ |  |  |  |  |  |
| ΔD_1st_%^†^ | 0.510 | 0.884 | 0.839 | 0.603 | 0.681 |
| TSP (non-SD vs SD) ^†^ | 0.980 | 0.256 | 0.610 | 0.917 | 0.649 |
| TNBC |  |  |  |  |  |
| ΔD_1st_%^†^ | 0.870 | 0.643 | 0.667 | 0.857 | 0.745 |
| TSP (non-SD vs SD) ^†^ | 0.957 | 0.464 | 0.595 | 0.929 | 0.686 |
| **2nd-timepoint Subgroup** |  |  |  |  |  |
| HR+/HER2- |  |  |  |  |  |
| ΔD_2nd_%^†^ | 0.889 | 0.518 | 0.163 | 0.978 | 0.553 |
| TSP (non-SD vs SD) ^†^ | 1.000 | 0.235 | 0.122 | 1.000 | 0.309 |
| HER2+ |  |  |  |  |  |
| ΔD_2nd_%^†^ | 0.909 | 0.467 | 0.714 | 0.778 | 0.730 |
| TSP (non-SD vs SD) ^†^ | 1.000 | 0.167 | 0.638 | 1.000 | 0.662 |
| TNBC |  |  |  |  |  |
| ΔD_2nd_%^†^ | 0.500 | 0.870 | 0.727 | 0.714 | 0.718 |
| TSP (non-SD vs SD) ^†^ | 1.000 | 0.174 | 0.457 | 1.000 | 0.513 |

TSP tumor shrinkage patterns, CS concentric shrinkage, DD diffuse decrease, DIO decrease of intensity only, SD stable disease, ΔD_early_% the percentage changes in tumor size after early MRI, ΔD_1st_% the percentage changes in tumor size at 1st-MRI, ΔD_2nd_% the percentage changes in tumor size at 2nd-MRI

^†^ Binary variable, the cut-off value was calculated using ROC curve with percentage changes in tumor size; Tumor shrinkage patterns were classified into two categories: non-SD (CS, DD, DIO) and SD

**Table S8** MRI-based tumor shrinkage patterns association with pCR according to different molecular subtypes in the subgroup analysis cohorts

| Shrinkage pattern | HR+/HER2- | | | | HER2+ | | | | TNBC | | | |
| --- | --- | --- | --- | --- | --- | --- | --- | --- | --- | --- | --- | --- |
|  | N | pCR | non-pCR | *p* | N | pCR | non-pCR | *p* | N | pCR | non-pCR | *p* |
| **1st-timepoint Subgroup** | 100 | 8 | 92 |  | 94 | 51 | 43 |  | 51 | 23 | 28 |  |
| CS | 45 (45%) | 3 (38%) | 42 (46%) | 0.167  (CS vs. DIO)  0.041  (CS vs. SD) | 56 (60%) | 36 (71%) | 20 (47%) | 0.167  (CS vs. DIO)  <0.001*  (CS vs. SD) | 32 (63%) | 19 (83%) | 13 (46%) | NA  (CS vs. DIO)  <0.001*  (CS vs. SD) |
| CS to small foci | 0 (0%) | 0 (0%) | 0 (0%) |  | 6 (6.4%) | 6 (12%) | 0 (0%) |  | 0 (0%) | 0 (0%) | 0 (0%) |  |
| simple CS | 43 (43%) | 3 (38%) | 40 (43%) |  | 44 (47%) | 27 (53%) | 17 (40%) |  | 31 (61%) | 18 (78%) | 13 (46%) |  |
| CS plus decreased enhancement | 2 (2.0%) | 0 (0%) | 2 (2.2%) |  | 6 (6.4%) | 3 (5.9%) | 3 (7.0%) |  | 1 (2.0%) | 1 (4.3%) | 0 (0%) |  |
| DD | 13 (13%) | 5 (63%) | 8 (8.7%) | 0.002*  (DD vs. CS)  <0.001*  (DD vs. SD) | 23 (24%) | 12 (24%) | 11 (26%) | 0.053  (DD vs. CS)  0.002*  (DD vs. SD) | 5 (10%) | 3 (13%) | 2 (7.1%) | 0.167  (DD vs. CS)  0.006*  (DD vs. SD) |
| CS with surrounding lesions | 4 (4.0%) | 0 (0%) | 4 (4.3%) |  | 6 (6.4%) | 2 (3.9%) | 4 (9.3%) |  | 1 (2.0%) | 1 (4.3%) | 0 (0%) |  |
| residual multinodular lesions | 9 (9.0%) | 5 (0%) | 4 (4.3%) |  | 17 (18%) | 10 (20%) | 7 (16%) |  | 4 (7.8%) | 2 (8.7%) | 2 (7.1%) |  |
| DIO | 2 (2.0%) | 0 (0%) | 2 (2.2%) | 0.087  (DD vs. DIO) | 3 (3.2%) | 2 (3.9%) | 1 (2.3%) | 0.167  (DD vs. DIO) | 0 (0%) | 0 (0%) | 0 (0%) | NA  (DD vs. DIO) |
| SD | 40 (40%) | 0 (0%) | 40 (43%) | NA  (SD vs. DIO) | 12 (13%) | 1 (2.0%) | 11 (26%) | 0.014  (SD vs. DIO) | 14 (27%) | 1 (4.3%) | 13 (46%) | NA  (SD vs. DIO) |
| **2nd-timepoint Subgroup** | 94 | 9 | 85 |  | 74 | 44 | 30 |  | 39 | 16 | 23 |  |
| CS | 50 (53%) | 3 (33%) | 47 (55%) | 0.167  (CS vs. DIO)  0.092  (CS vs. SD) | 37 (50%) | 23 (52%) | 14 (47%) | 0.167  (CS vs. DIO)  0.002*  (CS vs. SD) | 24 (62%) | 12 (75%) | 12 (52%) | NA  (CS vs. DIO)  0.019  (CS vs. SD) |
| CS to small foci | 1 (1.1%) | 1 (11%) | 0 (0%) |  | 5 (6.8%) | 5 (11%) | 0 (0%) |  | 3 (7.7%) | 3 (19%) | 0 (0%) |  |
| simple CS | 48 (51%) | 2 (22%) | 46 (54%) |  | 27 (36%) | 14 (32%) | 13 (43%) |  | 19 (49%) | 8 (50%) | 11 (48%) |  |
| CS plus decreased enhancement | 1 (1.1%) | 0 (00%) | 1 (1.2%) |  | 5 (6.8%) | 4 (9.1%) | 1 (3.3%) |  | 2 (5.1%) | 1 (6.3%) | 1 (4.3%) |  |
| DD | 22 (23%) | 6 (67%) | 16 (19%) | 0.003*  (DD vs. CS)  0.004*  (DD vs. SD) | 30 (41%) | 20 (45%) | 10 (33%) | 0.133  (DD vs. CS)  0.002*  (DD vs. SD) | 11 (28%) | 4 (25%) | 7 (30%) | 0.075  (DD vs. CS)  0.086  (DD vs. SD) |
| CS with surrounding lesions | 5 (5.3%) | 3 (33%) | 2 (2.4%) |  | 7 (9.5%) | 4 (9.1%) | 3 (10%) |  | 5 (13%) | 2 (13%) | 3 (13%) |  |
| residual multinodular lesions | 17 (18%) | 3 (33%) | 14 (16%) |  | 23 (31%) | 16 (36%) | 7 (23%) |  | 6 (15%) | 2 (13%) | 4 (17%) |  |
| DIO | 2 (2.1%) | 0 (0%) | 2 (2.4%) | 0.167  (DD vs. DIO) | 2 (2.7%) | 1 (2.3%) | 1 (3.3%) | 0.167  (DD vs. DIO) | 0 (0%) | 0 (0%) | 0 (0%) | NA  (DD vs. DIO) |
| SD | 20 (21%) | 0 (0%) | 20 (24%) | NA  (SD vs. DIO) | 5 (6.8%) | 0 (0%) | 5 (17%) | 0.048  (SD vs. DIO) | 4 (10%) | 0 (0%) | 4 (17%) | NA  (SD vs. DIO) |

pCR pathologic complete response, HER2 human epidermal growth factor receptor 2, TNBC triple-negative breast cancer, HR hormone receptor, CS concentric shrinkage, DD diffuse decrease, DIO decrease of intensity only, SD stable disease, NA not applicable

* Following adjustment for multiple comparisons with Bonferroni’s correction, the statistically significant *p* values were annotated (*p* < 0.00833)

**Table S9** Univariate and multivariate analysis of factors associated with pCR according to different molecular subtypes in the subgroup analysis cohorts

| Characteristics | 1st-timepoint Subgroup | | | | | | 2nd-timepoint Subgroup | | | | | |
| --- | --- | --- | --- | --- | --- | --- | --- | --- | --- | --- | --- | --- |
|  | Univariate analysis | | | Multivariate analysis | | | Univariate analysis | | | Multivariate analysis | | |
|  | OR | 95%CI | *p* | OR | 95%CI | *p* | OR | 95%CI | *p* | OR | 95%CI | *p* |
| **HR+/HER2-** |  |  |  |  |  |  |  |  |  |  |  |  |
| Tumor size (mm) |  |  |  |  |  |  |  |  |  |  |  |  |
| D_pre_* | 1.00 | 0.97-1.03 | 0.995 | - | - | - | 0.98 | 0.94-1.03 | 0.462 | - | - | - |
| D_1st/2nd_* | 0.99 | 0.95-1.03 | 0.655 | - | - | - | 0.96 | 0.91-1.02 | 0.153 | - | - | - |
| ΔD_1st_/ΔD_2nd_%^†^ | 1.61 | 1.01-2.58 | 0.048 | 0.87 | 0.46-1.64 | 0.659 | 1.68 | 1.11-2.53 | 0.014 | 1.61 | 1.01-2.59 | 0.046 |
| Shrinkage pattern |  |  |  |  |  |  |  |  |  |  |  |  |
| CS | Ref | Ref | Ref | Ref | Ref | Ref | Ref | Ref | Ref | Ref | Ref | Ref |
| DD | 8.75 | 1.73-44.16 | 0.009 | 9.99 | 1.78-56.04 | 0.009 | 5.88 | 1.31-26.27 | 0.020 | 7.72 | 1.55-38.53 | 0.013 |
| DIO | NA | NA | NA | NA | NA | NA | NA | NA | NA | NA | NA | NA |
| SD | NA | NA | NA | NA | NA | NA | NA | NA | NA | NA | NA | NA |
| **HER2+** |  |  |  |  |  |  |  |  |  |  |  |  |
| Tumor size (mm) |  |  |  |  |  |  |  |  |  |  |  |  |
| D_pre_* | 0.99 | 0.96-1.01 | 0.268 | - | - | - | 0.98 | 0.96-1.01 | 0.168 | - | - | - |
| D_1st/2nd_* | 0.97 | 0.94-1.00 | 0.025 | - | - | - | 0.96 | 0.93-0.99 | 0.019 | - | - | - |
| ΔD_1st_/ΔD_2nd_%^†^ | 1.86 | 1.35-2.57 | <0.001 | 1.75 | 1.20-2.56 | 0.004 | 1.45 | 1.10-1.91 | 0.008 | - | - | - |
| Shrinkage pattern |  |  |  |  |  |  |  |  |  |  |  |  |
| CS | Ref | Ref | Ref | Ref | Ref | Ref | Ref | Ref | Ref | - | - | - |
| DD | 0.61 | 0.23-1.62 | 0.319 | 0.71 | 0.25-2.05 | 0.531 | 1.22 | 0.44-3.34 | 0.702 | - | - | - |
| DIO | 1.11 | 0.10-13.03 | 0.933 | 4.91 | 0.34-70.27 | 0.241 | 0.61 | 0.04-10.53 | 0.733 | - | - | - |
| SD | 0.05 | 0.01-0.42 | 0.006 | 0.19 | 0.02-1.90 | 0.159 | NA | NA | NA | - | - | - |
| **TNBC** |  |  |  |  |  |  |  |  |  |  |  |  |
| Tumor size (mm) |  |  |  |  |  |  |  |  |  |  |  |  |
| D_pre_* | 1.00 | 0.97-1.03 | 0.963 | - | - | - | 0.99 | 0.96-1.02 | 0.484 | - | - | - |
| D_1st/2nd_* | 0.98 | 0.94-1.01 | 0.186 | - | - | - | 0.97 | 0.93-1.02 | 0.226 | - | - | - |
| ΔD_1st_/ΔD_2nd_%^†^ | 1.94 | 1.29-2.92 | 0.001 | 1.62 | 0.98-2.68 | 0.061 | 1.43 | 1.03-1.98 | 0.033 | - | - | - |
| Shrinkage pattern |  |  |  |  |  |  |  |  |  |  |  |  |
| CS | Ref | Ref | Ref | Ref | Ref | Ref | Ref | Ref | Ref | - | - | - |
| DD | 1.03 | 0.15-7.02 | 0.979 | 2.08 | 0.24-18.02 | 0.506 | 0.57 | 0.13-2.48 | 0.454 | - | - | - |
| DIO | NA | NA | NA | NA | NA | NA | NA | NA | NA | - | - | - |
| SD | 0.05 | 0.01-0.45 | 0.007 | 0.19 | 0.02-2.36 | 0.197 | NA | NA | NA | - | - | - |

pCR pathologic complete response, HER2 human epidermal growth factor receptor 2, TNBC triple-negative breast cancer, HR hormone receptor, CS concentric shrinkage, DD diffuse decrease, DIO decrease of intensity only, SD stable disease, OR odds ratio, Ref reference, NA not applicable, D_pre_ the tumor size at Pre-MRI, D_1st_ the tumor size at 1st-MRI, D_2nd_ the tumor size at 2nd-MRI, ∆D_1st_/∆D_2nd_% the percentage changes in tumor size at 1st-MRI or 2nd-MRI (continuous variable for 10% increment)

*D_pre_, D_1st_ and D_2nd_ were analyzed only in univariate analysis; ^†^ΔD_1st_/ΔD_2nd_% with higher OR was used in multivariate analysis; NA was due to the fact that the shrinkage pattern had zero samples in either the pCR or non-pCR group.


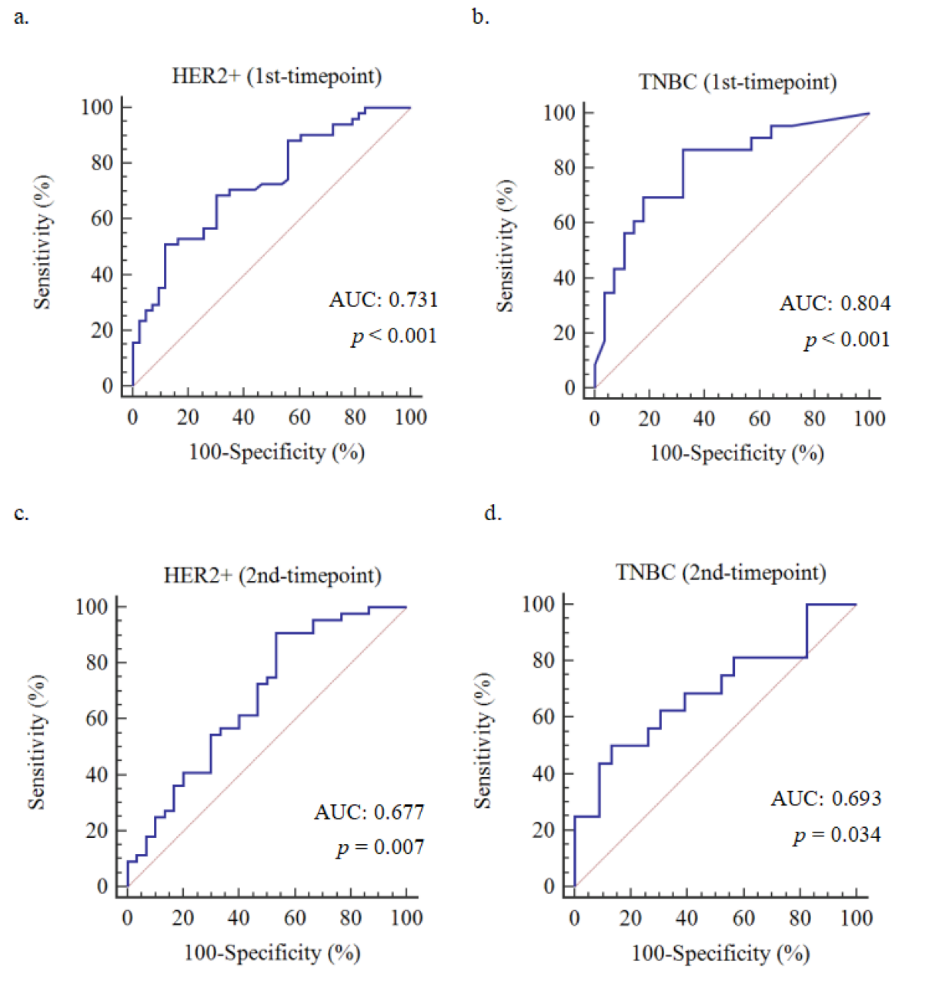


**Fig. S1** Receiver operating characteristic (ROC) curves of the change in tumor size (continuous variable) at 1st-timepoint and 2nd-timepoint for pathologic complete response (pCR) prediction in the breast. (a) ROC curve of the change in tumor size at 1st-timepoint for pCR prediction in HER2+ subtype. (b) ROC curve of the change in tumor size at 1st-timepoint for pCR prediction in TNBC. (c) ROC curve of the change in tumor size at 2nd-timepoint for pCR prediction in HER2+ subtype. (d) ROC curve of the change in tumor size at 2nd-timepoint for pCR prediction in TNBC.
